# Clinical significance and potential mechanism of heat shock factor 1 in acute myeloid leukemia

**DOI:** 10.18632/aging.204267

**Published:** 2022-09-06

**Authors:** Chunyi Lyu, Qian Wang, Xuewei Yin, Zonghong Li, Teng Wang, Yan Wang, Siyuan Cui, Kui Liu, Zhenzhen Wang, Chang Gao, Ruirong Xu

**Affiliations:** 1 First Clinical Medical College, Shandong University of Traditional Chinese Medicine, Jinan, People's Republic of China; 2 College of Traditional Chinese Medicine, Shandong University of Traditional Chinese Medicine, Jinan, People's Republic of China; 3 Shandong Key Laboratory of Hematology of Integrated Traditional Chinese and Western Medicine of Health Commission, Institute of Hematology of Shandong University of Traditional Chinese Medicine, Jinan, People's Republic of China; 4 Department of Hematology, Affiliated Hospital of Shandong University of Traditional Chinese Medicine, Jinan, People's Republic of China

**Keywords:** acute myeloid leukemia, heat shock factor 1, HSF1, prognosis, biomarker

## Abstract

Background: Heat shock factor 1 (HSF1) is now considered to have the potential to be used as a prognostic biomarker in cancers. However, its clinical significance and potential function in acute myeloid leukemia (AML) remain underexplored.

Methods: In this study, the expression pattern and clinical significance of HSF1 in AML were examined by integrating data from databases including The Cancer Genome Atlas (TCGA), The Genotype–Tissue Expression (GTEx), Vizome, Cancer Cell Line Encyclopedia (CCLE) and Gene Expression Omnibus (GEO). Linkedomics was applied to collect HSF1–related genes in AML. GeneMANIA was applied to outline HSF1–related functional networks. CancerSEA analysis, Kyoto Encyclopedia of Genes and Genomes (KEGG) analysis and Gene Set Enrichment Analysis (GSEA) were performed to mine the potential mechanism of HSF1 in leukemogenesis. Single–sample Gene Set Enrichment Analysis (ssGSEA) was applied to explore the correlation between HSF1 and infiltrating immune cells in AML.

Results: HSF1 expression was elevated in AML compared to healthy controls and indicate a poor overall survival. HSF1 expression was significantly correlated with patients age, associated with patient survival in subgroup of bone marrow blasts (%) >20. Functional analyses indicated that HSF1 plays a role in the metastatic status of AML, and is involved in inflammation–related pathways and biological processes. HSF1 expression was significantly correlated with the immune infiltration of nature killer cells and T cell population.

Conclusion: HSF1 plays a vital role in the molecular network of AML pathogenesis, and has the potential to be a biomarker for prognosis prediction.

## INTRODUCTION

Acute myeloid leukemia (AML) is a highly malignant disease with a poor survival rate despite continuous advances in treatment [1]. Advances in molecular biology and multiomics technology have increased the knowledge of how genes control physiological functions involved in the modulation of specific phenotypes and drug efficacy, and provide highly informative data for studying the molecular state of disease biology [2, 3]. Increasingly omics data have promoted the exploration of the interconnections across molecular layers as well as investigation of AML pathogenesis and identification of underlying diagnostic and therapeutic molecules.

Heat shock factors (HSFs) are a class of evolutionarily conserved transcription factors, that regulate the heat shock response (HSR) by mediating the expression pattern of heat shock proteins (HSPs) [4, 5]. HSPs are observed to be increased in a wide variety of cancers and are involved in almost every aspect of cancer development [6, 7]. HSP90 overexpression has been reported in AML, and HSP90 inhibitors have been confirmed to be effective agents against primary AML blasts [8]. The transcriptional activation of the HSPs is under the control of HSFs. Four HSFs HSF1, HSF2, HSF4 and HSF5, exist in humans, and HSF1 is the primary activator in human tissues [9]. HSF1, localized to chromosome 8q24.3, prevents cancer cell death when exposed to stressors, which indispensable for various malignant transformation–related signaling pathways, thereby facilitating cancer cell proliferation and migration and affecting the tumor microenvironment [10]. Additionally, significant correlations between HSF1 and immune checkpoint genes and the levels of B cells, CD8+ T cells, CD4+ T cells, macrophages, neutrophils, and dendritic cells have been detected in multiple kinds of cancers [11]. There are studies showing that AML molecular features correlate with clinicopathological characteristics and prognosis [12]. Emerging evidence has demonstrated that the upregulation of HSF1 is associated with poor overall survival (OS) in multiple cancers, suggesting that HSF1 has the potential to serve as an independent prognostic biomarker [13–15].

Previous research on HSP expression from both the stress response and cancer fields involves HSF1. A study confirmed that HSF1 is elevated in AML cell lines including HL–60 and OCI–AML3, acting as a promoter of cell proliferation [16]. Additionally, the expression level of HSF1 was observed to be enhanced in chronic lymphocytic leukemia (CLL) and decreased in patients responding to in the rituximab–bendamustine protocol [17], which supports its potential to be a biomarker for efficacy prediction. These results demonstrated that HSF1 is involved in leukemogenesis and has the potential to be a novel biomarker but its clinical significance and potential function in AML have not been fully investigated.

In this study, the expression level, diagnostic efficiency, and prognostic significance of HSF1 in AML, as well as the correlations between HSF1 and clinical parameters, were investigated and validated based on The Cancer Genome Atlas (TCGA), Genotype–Tissue Expression (GTEx), Gene Expression Omnibus (GEO), Cancer Cell Line Encyclopedia (CCLE), and Beat AML data viewer (Vizome). Then, the potential mechanism of HSF1 in AML was explored based on annotation of transcriptome and single–cell sequencing data. The molecular regulatory association between HSF1 and its related genes was explored by constructing a protein–protein interaction network. Additionally, the correlation between HSF1 expression and immune cell subset abundance was further explored. This study aimed to elucidate the role of HSF1 in AML at a systems level in order to explore the clinical implications of HSF1 and the relevant molecular mechanisms in AML pathogenesis.

## RESULTS

### HSF1 expression is elevated in AML and indicates poor prognosis

**Figure 1:**
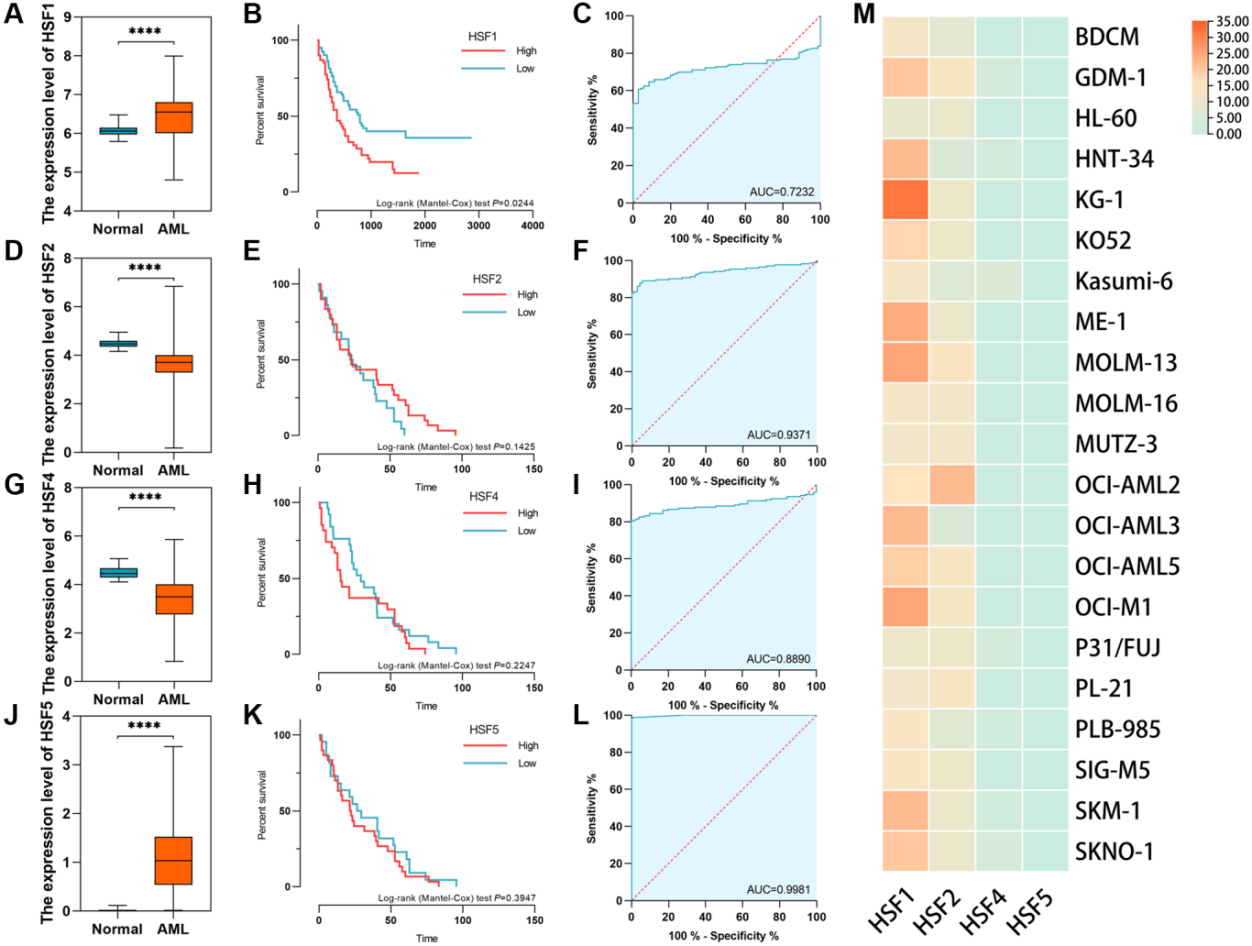
**The expression level, diagnostic efficacy and prognostic significance of HSF family in acute myeloid leukemia (AML) based on data from the cancer genome atlas (TCGA) and the genotype–tissue expression (GETx) processed using the TOIL pipeline.** The validation datasets were downloaded from the Gene Expression Omnibus (GEO), and Cancer Cell Line Encyclopedia (CCLE). (**A**–**C**) The expression level, diagnostic efficacy and prognostic significance of HSF1 in AML. (**D**–**F**) The expression level, diagnostic efficacy and prognostic significance of HSF2 in AML. (**G**–**I**) The expression level, diagnostic efficacy and prognostic significance of HSF4 in AML. (**J**–**L**) The expression level, diagnostic efficacy and prognostic significance of HSF5 in AML. (**M**) The expression level of HSFs examined in leukemia cell lines. ^****^P < 0.001.

**Figure 2:**
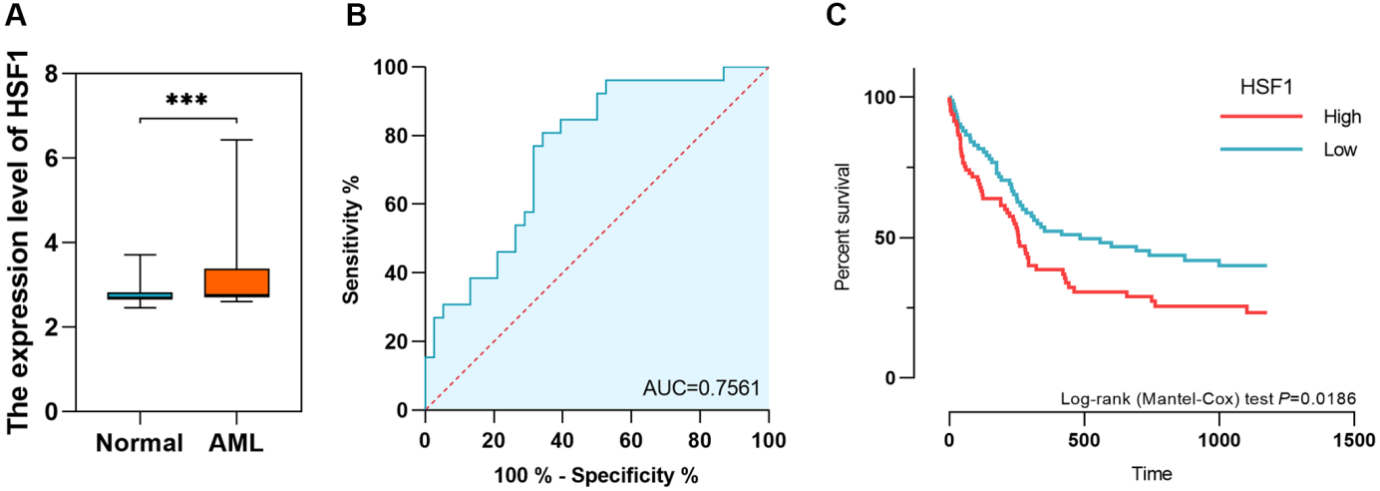
**The expression level, diagnostic value and prognostic significance of HSF1 in AML based on data from gene expression omnibus (GEO).** (**A** and **B**) The expression level and diagnostic value of HSF1 in AML based on data from GSE9476. (**C**) The prognostic significance of HSF1 based on data from GSE12417. ^***^P < 0.01.

The HSF family in humans includes HSF1, HSF2, HSF4 and HSF5. According to data from AML based on TCGA and healthy controls derived from GTEx, HSF1 and HSF5 were elevated, while HSF2 and HSF3 were decreased in AML compared to healthy controls (Figure 1A, 1D, 1J and 1G). Additionally, HSF1 was highly expressed in most of 21 leukemia cell lines, especially the acute myeloid leukemia cell line KG–1 (Figure 1M). The receiver operating characteristic (ROC) curve analyses showed good discriminative ability of HSF1, area under the curve (AUC) is 0.723, (Figure 1C), HSF2 (AUC = 0.737), HSF4 (AUC = 0.889), and HSF5 (AUC = 0.998) (Figure 1F, 1I, and 1L). There were statistically significant changes in the expression of all HSF members, but only HSF1 had marked prognostic significance (Figure 1B, 1E, 1H and 1K). A high expression level of HSF1 predicts poor OS, which supports the idea that HSF1 is a prognostic marker for poor outcomes. The expression level and diagnostic efficacy were validated in replication datasets. In GSE9476, HSF1 was validated to be highly expressed in 26 AML patients compared to 38 healthy controls, and AUC was 0.756 (Figure 2A and 2B). The association of high HSF1 expression with a poor prognosis for AML was validated using GSE12417, and data from 163 patients were included (Figure 2C).

### Clinical significance of HSF1 expression in AML

**Figure 3:**
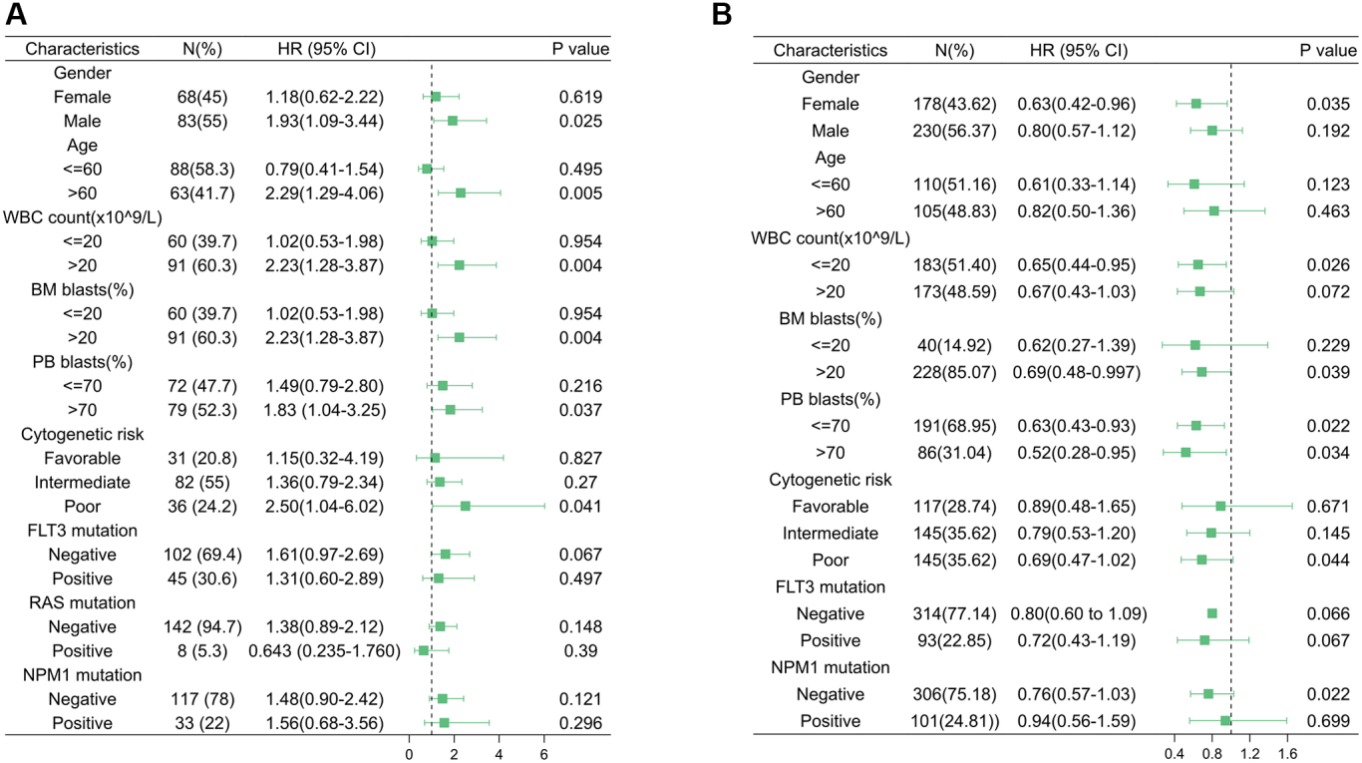
**Acute myeloid leukemia subgroup overall survival analyses.** Forest plot of Cox analyses of HSF1 for different subgroups based on data from The Cancer Genome Atlas (TCGA) (**A**) and Vizome (**B**).

A logistic model was constructed to explore the association between HSF1 expression and clinical parameters of AML patients, and the results showed that HSF1 is significantly associated with patient age and peripheral blood (PB) blast proportion based on data from TCGA. Evidence from the Vizome indicated that HSF1 is significantly associated with patient age (Table 1). In both TCGA and Vizome, age was a significant associated factor for HSF1, but HSF1 bore no relationship to survival in the age subgroup. In subgroup of bone marrow (BM) blast proportion (%) >20, HSF1 was significantly associated with patient survival based on data from TCGA, which was also validated in the replication cohort based on data from Vizome (Figure 3).

**Table 1:** Association between HSF1 expression and clinical parameters.

**Characteristics**	**TCGA (*n* = 149)**		**LAML (*n* = 445)**	
	**OR**	***P* value**	**OR**	***P* value**
Gender	1.140 (0.600–2.171)	0.689	1.104 (0.650–1.875)	0.713
Age	2.000 (1.041–3.894)	0.039	1.027 (1.011,1.043)	0.001
WBC count (×10^9^/L)	1.306 (0.688–2.491)	0.414	1.002 (0.996–1.007)	0.573
BM blast proportion (%)	1.276 (0.664–2.461)	0.465	0.994 (0.982–1.007)	0.364
PB blast proportion (%)	2.722 (1.419–5.316)	0.003	1.002 (0.990–1.014)	0.740
Cytogenetic risk (Poor vs. Favorable and Intermediate)	1.843 (0.865–4.035)	0.117	0.786 (0.433–1.427)	0.429
FLT3 mutation (Positive vs. Negative)	1.755 (0.866–3.621)	0.122	0.825 (0.433–1.573)	0.560
NPM1 mutation (Positive vs. Negative)	1.044 (0.481–2.277)	0.912	0.757 (0.386–1.486)	0.429

### Functional analyses of HSF1 in AML

**Figure 4:**
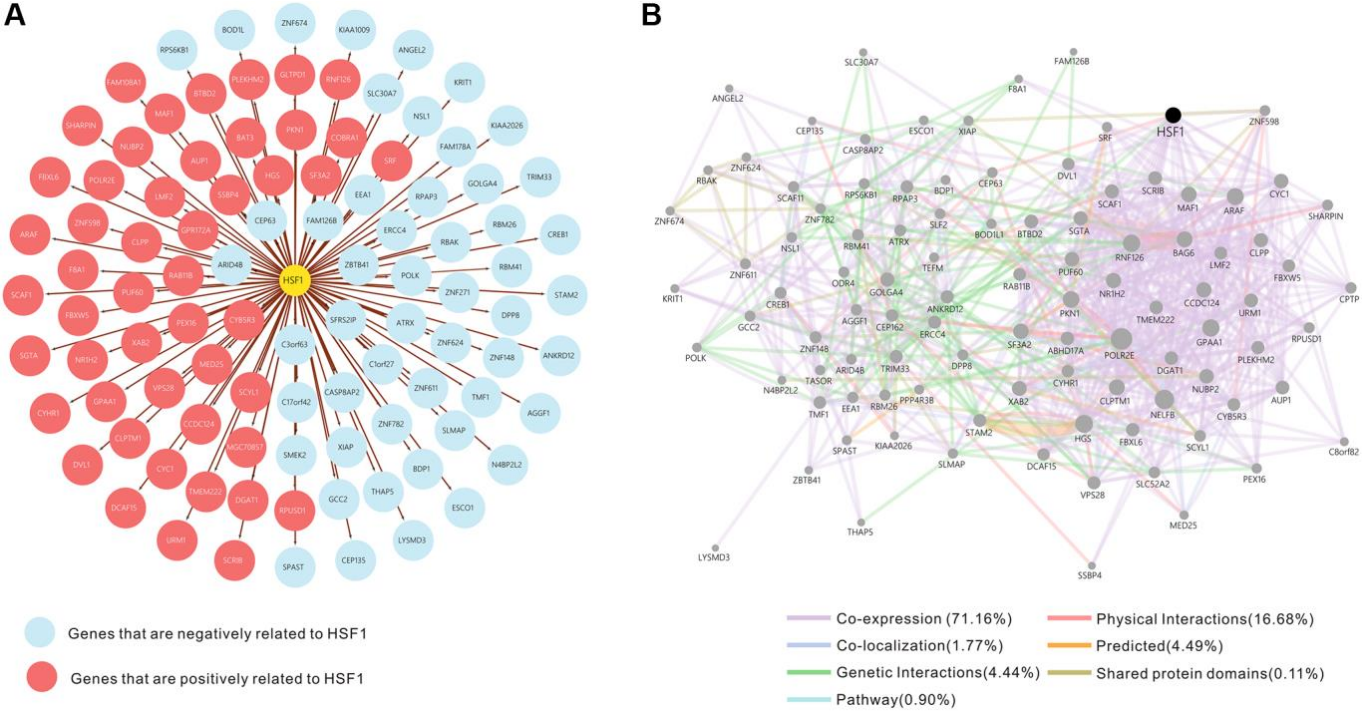
**The fifty genes with the strongest positive and negative correlations with HSF1.** (**A**) Network of HSF1 and correlated genes. Positively correlated genes are marked in red. Negatively correlated genes are marked in blue. (**B**) Network analysis of HSF1 and correlated genes using GeneMANIA.

**Figure 5:**
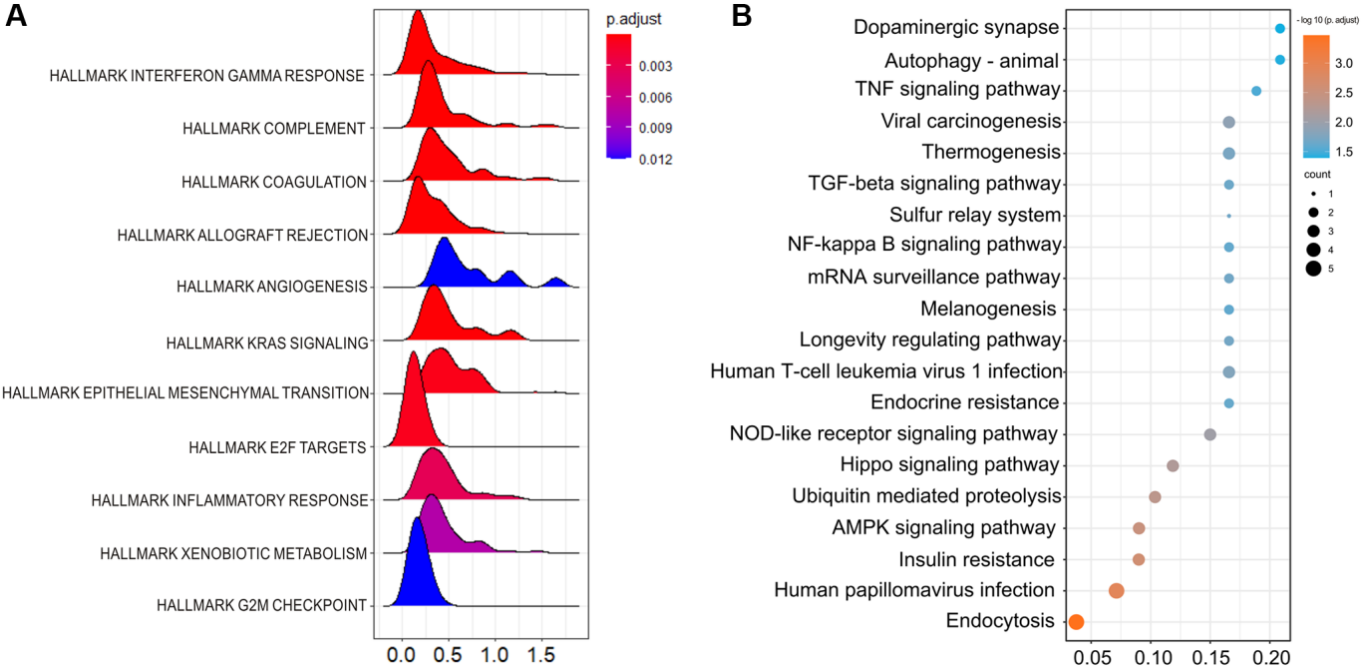
**The underlying mechanisms related to HSF1 are shown.** (**A**) Gene Set Enrichment Analysis (GSEA) of the deferentially expressed genes between the HSF1 high–expression versus HSF1 low–expression group in AML. (**B**) Kyoto Encyclopedia of Genes and Genomes (KEGG) enrichment analysis of HSF1 related genes.

**Figure 6:**
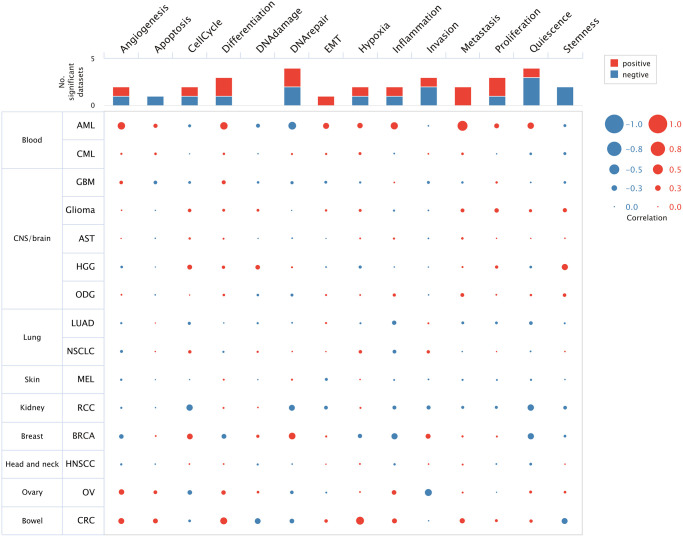
The function of HSF1 was analyzed at single–cell level based on the CancerSEA database.

The top 50 negatively and positively correlated genes related to HSF1 expression in AML were obtained from linkedomics (Figure 4A). Correlation analyses of HSF1–associated genes were conducted using GeneMANIA, and the results showed that 71.16% are annotated as co–expressed, 16.68% are annotated as physical interactions, 4.44% are annotated as genetic interactions, 1.77% are annotated as co–localization and 0.11% of genes are shared protein domains (Figure 4B). Kyoto Encyclopedia of Genes and Genomes (KEGG) pathway enrichment analyses of HSF1–associated genes showed that HSF1 is involved in the AMPK signaling pathway, Hippo signaling pathway, NF–kappa B signaling pathway, TGF–βsignaling pathway, and TNF signaling pathway (Figure 5B). CancerSEA analyses showed that the functional phenotypes of HSF1 in AML were significantly positively correlated with metastasis, angiogenesis, and differentiation inflammation and negatively correlated with DNA repair (Figure 6). The Gene Set Enrichment Analysis (GSEA) results showed that HSF1 was significantly related to HALLMARK INFLAMMATORY RESPONSE, HALLMARK INTERFERON GAMMA RESPONSE, HALLMARK ANGIOGENESIS, and HALLMARK KRAS SIGNALING in AML (Figure 5A).

### Immune infiltration analyses

**Figure 7:**
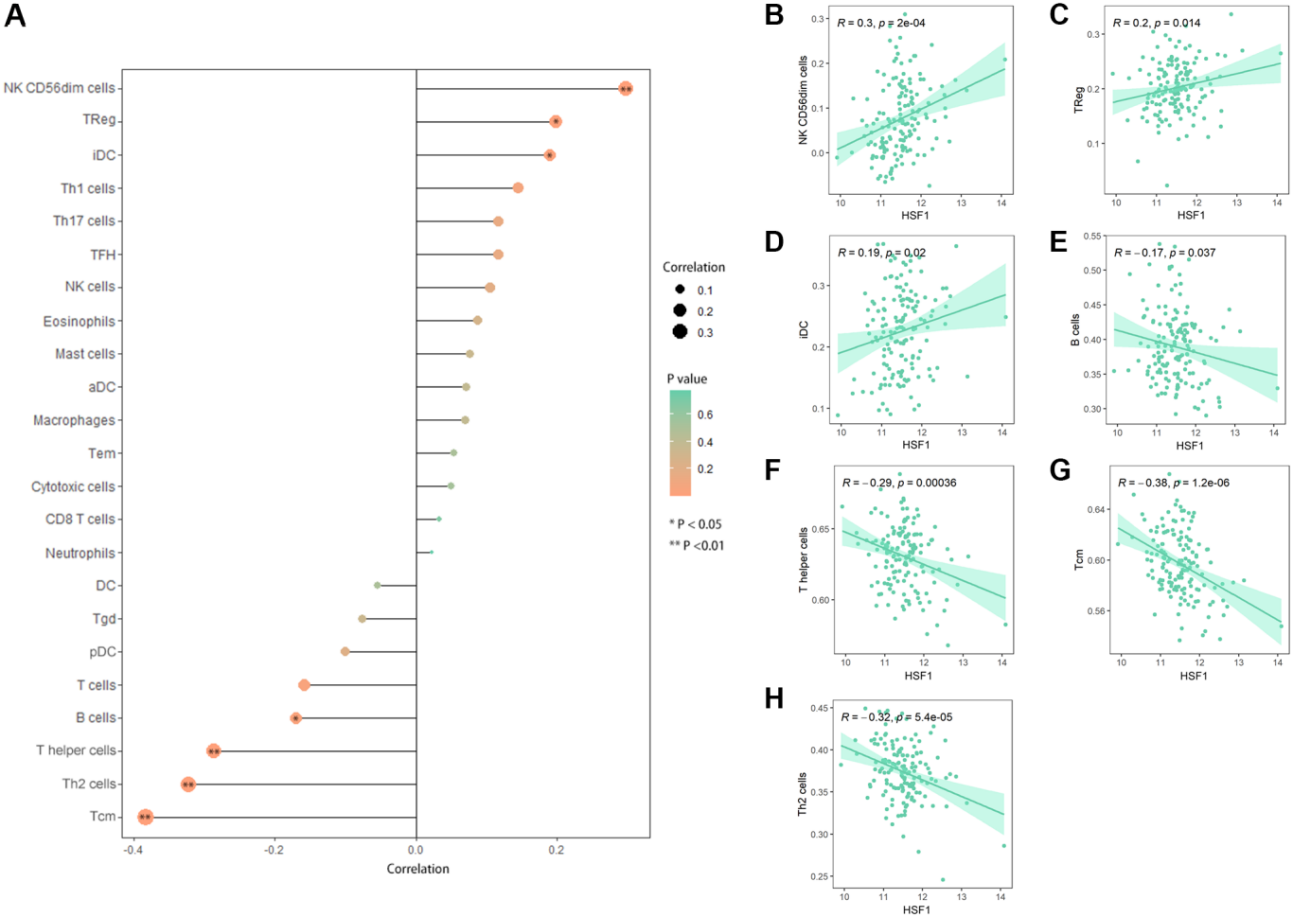
**Correlation analyses between HSF1 and immune infiltration.** (**A**) Lollipop plot showing the expression of HSF1 exhibits a strong correlation with the infiltration abundance of NK CD56dim cells (**B**), Tregs (**C**), immature DCs (iDCs) (**D**), B cells (**E**), T helper (Th) cells (**F**), T central memory (Tcm) cells (**G**) and Th2 cells (**H**). ^*^P < 0.05, ^**^P < 0.01.

Based on single–sample Gene Set Enrichment Analysis (ssGSEA), correlation between HSF1 expression and immune cell infiltration was analyzed. HSF1 expression was significantly related to the abundance of infiltrating immune cells in AML including the T cell population, nature killer (NK) cells, B cells, and immature dendritic cells (iDCs) (Figure 7A). There were significantly correlations between HSF1 expression and infiltrating levels of NK CD56dim cells (Figure 7B), Tregs (Figure 7C), iDCs (Figure 7D), B cells (Figure 7E), T helper (Th) cells (Figure 7F), T central memory (Tcm) cells (Figure 7G) and Th2 cells (Figure 7H).

## DISCUSSION

HSF–induced upregulation of HSPs is a key part of HSR. Among members of the HSF family, HSF1 is reported to be the major regulator of increased HSPs in cancer, and switches on during the development of cancer [18]. Generally, elevated expression of HSF1 has been described in various cancer types [11]. In hematological malignancies, the role of HSF1 has also been partially explored. HSF1 is elevated in Hodgkin lymphoma, multiple myeloma, CLL and AML [16, 19–21]. In multiple myeloma, HSF1 expression has been demonstrated to be related to poor prognosis [19]. Additionally, overexpression of HSF1 in CLL patients is associated with poor prognosis and localized in the nucleus of leukemic B cells [20]. In addition to being highly expressed in AML patient blood samples and AML cell lines, HSF1–pSer326 has been reported to predict the response to bortezomib–containing chemotherapy in pediatric AML [22].

HSF family members expressed in humans include HSF1, HSF2, HSF4 and HSF5, of which expression pattern, diagnostic efficiency and prognostic significance were investigated in this study. HSF1 and HSF5 were highly expressed, while HSF2 and HSF4 were decreased in AML compared to healthy controls, and elevated expression of HSF1 has prognostic significance, indicating a worse survival probability, which has the potential to be a prognosis–predicted biomarker. Further logistic analysis showed that HSF1 expression is correlated to patient age. Previous studies have suggested that elevated HSF1 increases both stress tolerance and lifespan of *C. elegans* [23, 24]. HSF1 can affect the aging process by upregulating HSP genes, modulating other stress/repair pathways, and influencing signaling systems [25]. Although HSF1 appears to be intimately connected to senescence pathways, the prognostic significance of HSF1 in elderly AML patients remains to be established. Additionally, survival analyses demonstrated that HSF1 predict poor prognosis in BM blast proportion (%) >20 subgroups. Leukemia is characterized by over production of abnormal blast cells in the bone marrow that often spill out into other hematopoietic tissues. The BM blast proportion reflects the malignancy of AML. The result indicated HSF1 might be a prognostic marker in higher malignancy.

It is known that HSF1 exerts its pro–oncogenic effects by mediating HSR–related biological processes in the oncogenic network. To further explore the underlying role of HSF1 in AML pathogenesis, multi-omics analyses were integrated in this study. In the results from single–cell sequencing data form CancerSEA, HSF1 was significantly related to various aspects of leukemogenesis, and a remarkable association was observed for HSF1 and leukemia stem cell metastasis in AML. The correlation between HSF1 expression and metastatic potential has been reported in breast, colon, lung, prostate, hepatocellular tumors and malignant melanoma, indicating that HSF1 supports anchorage–independent growth [26]. In AML, HSF1 may contribute to leukemia stem cells metastasis and is associated with malignant characteristic. Additionally, the functional phenotype of HSF1 is highly implicated in inflammation–related signaling pathways according to the results from the CancerSEA, GSEA and KEGG analyses. Evidence from previous studies has shown that HSF1 is widely involved in immune modulation. HSF1 works as an innate repressor of human immunodeficiency virus–induced inflammation by regulating NF–κB [27]. HSF1 was remarkably correlated with the levels of infiltrating cells and immune checkpoint genes in various cancers [11]. The clinical data indicate that AML risk was significantly associated with multiple autoimmune diseases, and inflammation may impact prognosis in AML patients [28, 29]. Additionally, inflammatory signaling pathways including TNFa/NF–κB, nod–like receptor signaling, TGF–β signaling and the interleukin family have been noted in AML and associated with leukemogenesis [30]. To further explore the correlations between the inflammation state and HSF1, ssGSEA was used to evaluate infiltrated immune cells and HSF1 expression in AML, and the results showed that HSF is significantly correlated with NK cells and T cells. Tumor cells are under the surveillance of immune cells, and disorders of NK cells and T cells–mediated immune responses contribute to leukemogenesis [31, 32]. HSF1 is necessary for optimal T cell proliferation at normal temperatures in the presence of robust stimuli and serves as the predominant regulator of the heat shock response, which represses the cytotoxicity of human NK cells *in vitro* and *in vivo* [33, 34]. HSF1 may affect immune cells in order to regulate the immune state in AML and influence leukemia cell growth, metastasis, and AML patient survival, but its mechanisms remain to be validated *in vitro* and *in vivo*.

In the present study, we integrate data from several sources of publicly available databases and aimed to systematically investigate the expression and functional roles of HSF1 in AML as well as its clinical implications. The results analyzed based on TCGA were validated using independent datasets sourced from GEO and Vizome. The credibility of the prediction was improved, but some inconsistency exists between different cohorts. Validated results provide an important perspective for understanding the potential role of HSF1 in AML, and contradictory or conflicting results represent open questions for future studies. Additionally, datasets are limited in diversity and sample sizes, large–scale prospective cohort studies should be implemented to warrant the screening value of HSF1 for clinical purposes.

In summary, HSF1 was screened as a potential diagnostic and prognostic biomarker for AML patients. The leukemogenesis mechanisms of HSF1 are a multifactor regulatory process that is involved in inflammatory states, leukemia stem cell metastasis and related signaling pathways. This study comprehensively elucidated the clinical significance and potential functions of HSF1, which provides a perspective to understand the complex molecular network of leukemogenesis.

## MATERIALS AND METHODS

### Data sources

In this study, mRNA sequencing data for AML and healthy controls were downloaded from UCSC XENA (https://xenabrowser.net/datapages/), which were sourced from the TCGA and GETx databases and processed using the Toil pipeline to minimize batch effects [35]. The validation datasets were downloaded from the GEO, CCLE (https://www.broadinstitute.org/ccle) [36], and Vizome (http://vizome.org/) [37].

### HSF expression, diagnostic, and prognostic analyses

The HSF family includes HSF1, HSF2, HSF4 and HSF5. The expression level, diagnostic efficacy and prognostic significance of HSFs in AML were detected based on the data sourced from TCGA and GTEx. Expression levels were analyzed for normality using the Kolmogorov–Smirnov test. An unpaired *t* test was used for independent samples, and the Mann–Whitney test was used if the assumptions of normality for the *t* tests were not fulfilled. The expression levels of HSF1, HSF2, HSF4, and HSF5 in AML cell lines was further examined in CCLE. The ROC curve was then generated to evaluate the diagnostic value of HSF1 in AML based on data from TCGA. An AUC closer to 1 means better classification of the samples, and indicates a test of perfect diagnostic value, whereas an AUC of 0.5 indicated no diagnostic value.

High (top 50%) and low (bottom 50%) HSF1 expression groups were divided according to the median expression of HSF1. OS of the high expression group and the low expression group was assessed using the Kaplan-Meier method by log-rank test based on data sourced from TCGA. Subsequently, AML patients were divided into different subgroups based on clinical variables, and the prognostic value of HSF1 among different subgroups was assessed to further elucidate the prognostic significance of HSF1 in AML patient subgroups.

HSF1 expression level and diagnostic efficacy were validated using GSE9476, and the prognostic significance was validated using GSE12417 and Vizome. Statistical analyses and visualization were performed using GraphPad Prism (version 8.0, GraphPad Software Inc., La Jolla, CA, USA), and a *P*-value < 0.05 was considered to be statistically significant.

### Evaluation of correlation between HSF1 and clinical parameters in AML

The association of HSF1 expression with clinical parameters including patient gender, age, BM blast proportion, PB blast proportion, white blood cell (WBC) counts, cytogenetic risks, NPM1 mutation and FLT3 mutation was estimated using logistic regression models based on TCGA data, which was validated using data sourced from the Vizome database. This analysis was conducted using IBM SPSS Statistics (version 26.0, SPSS Inc., Chicago, IL, USA) by taking HSF1 as an independent variable, and relevant clinical parameters were set as dependent variables. Clinical parameters with a threshold *P*-value < 0.05 were identified as independent clinical factors associated with HSF1 expression.

### CancerSEA analyses

CancerSEA (http://biocc.hrbmu.edu.cn/CancerSEA/) is an integrative database depicts functional states of cancer cells at a single–cell resolution, covering 14 functional phenotypes of 41,900 cancer single cells from 25 cancer types [38]. In order to explore the underlying role of HSF1 in leukemogenesis, CancerSEA was used for functional analysis based on single–cell level. The correlation between HSF1 and functional states in AML, including invasion, metastasis, proliferation, angiogenesis, apoptosis, stemness, cell cycle, differentiation, DNA damage, DNA repair, hypoxia, epithelial–mesenchymal transition, inflammation and quiescence was investigated.

### Linkedomics analyses

LinkedOmics (http://www.linkedomics.org/login.php), a database containing multiomics data and clinical data for 32 cancer types and a total of 11,158 patients from TCGA project [39]. Genes related to HSF1 expression in AML were screened in linkedomics using a Pearson's correlation. The top 50 genes positively and negatively associated with HSF1 expression were collected for further analysis. Cytoscape software (version 3.9.1) was used for visualization [40].

### GeneMANIA analyses

GeneMANIA (http://www.genemania.org) is a tool for generating hypotheses about gene function, analyzing gene lists and prioritizing genes for functional assays [41]. Positively and negatively correlated genes were uploaded to GeneMANIA to determine protein–protein interactions, gene regulation, coexpression and pathway association.

### Functional enrichment analyses

To understand the functional implications of HSF1 in AML. KEGG analyses of positively and negatively correlated genes of HSF1 were performed based on KOBAS–i (http://kobas.cbi.pku.edu.cn/) [42]. Differentially expressed genes between high (top 50%) and low (bottom 50%) HSF1 expression groups were screen based on R package “DESeq2” (version 1.26.0). Based on the differential analyses of high and low HSF1 expression groups, the differentially expressed genes (DEGs) were sorted according to log fold change and enriched to carry out GSEA based on the R package “clusterProfiler” (version 3.14.3). The Hallmark list “h.all.v7.2.symbols.gmt” from MSigDB collections (version 5) was used as the gene set references. A Benjamini–Hochberg false discovery rate (FDR)-value < 0.05 and *P.* adjust value <0.05 were considered to be significantly enriched.

### Immune infiltration analyses

HSF1 expression with immune cell infiltration levels in AML was further explored. The gene markers of tumor–infiltrating immune cell markers are referenced in prior studies [43]. The infiltration levels of 23 immune cell types including DCs, immature DCs, activated DCs, plasmacytoid DCs, NK cells, NK CD56dim cells, B cells, T cells, CD8 T cells, Th cells, Tcm cells , T effector memory (Tem) cells, T follicular helper (TFH) cells, T gamma delta (Tgd) cells, Th1 cells, Th2 cells, Th17 cells, Treg cells, cytotoxic cells, eosinophils, macrophages, mast cells, and neutrophils, were quantified as enrichment scores using ssGSEA based on the R package “GSVA” (version 3.15), and the enrichment score represents the infiltration abundance of immune cells. A Pearson’s correlation was used to investigate the correlation between HSF1 expression and immune cell infiltration. A *P*-value < 0.05 was considered to be a significant association.

### Data availability

All R code used for the pre-processing of the data is available at Open Science Framework (OSF), https://osf.io/nw23m/. The data supporting the conclusions of this article are available upon request to the corresponding author.
